# MicroRNA engineered umbilical cord stem cell-derived exosomes direct tendon regeneration by mTOR signaling

**DOI:** 10.1186/s12951-021-00906-4

**Published:** 2021-06-05

**Authors:** Zhixiao Yao, Juehong Li, Hao Xiong, Haomin Cui, Jiexin Ning, Shikun Wang, Xingyu Ouyang, Yun Qian, Cunyi Fan

**Affiliations:** 1 Department of Orthopaedics, Shanghai Jiao Tong University Affiliated Sixth People’s Hospital, Shanghai, 200233 China; 2 Department of Plastics, Binzhou People’s Hospital, Binzhou, 256610 China

**Keywords:** Tendon repair, Human umbilical cord mesenchymal stem cell, Exosome, PTEN, mTOR, miR-29a-3p

## Abstract

**Background:**

Exosomes are extracellular vesicles of nano-structures and represent an emerging nano-scale acellular therapy in recent years. Tendon regeneration is a sophisticated process in the field of microsurgery due to its poor natural healing ability. To date, no successful long-term solution has been provided for the healing of tendon injuries. Functional recovery requires advanced treatment strategies. Human umbilical cord mesenchymal stem cell-derived exosomes (HUMSC-Exos) are considered as promising cell-free therapeutic agents. However, few studies reported their potential in the tendon repair previously. In this study, we explored the roles and underlying mechanisms of HUMSC-Exos in the tendon regeneration.

**Results:**

Expression of tendon‐specific markers in, and collagen deposition by, tendon-derived stem cells (TDSCs) treated with HUMSC-Exos increased in vitro. In a rat Achilles tendon injury model, treatment with HUMSC-Exos improved the histological structure, enhanced tendon-specific matrix components, and optimized biomechanical properties of the Achilles tendon. Findings in miRNA sequencing indicated a significant increase in miR-29a-3p in HUMSC-Exo-treated Achilles tendons. Next, luciferase assay in combination with western blot identified phosphatase and tensin homolog (PTEN) as the specific target of miR-29a-3p. Furthermore, we applied a miR-29a-3p-specific agonist to engineer HUMSC-Exos. These HUMSC-Exos overexpressing miR-29a-3p amplified the gain effects of HUMSC-Exos on tendon healing in vivo. To explore the underlying mechanisms, a transforming growth factor-β1 (TGF-β1) inhibitor (SB-431542), mTOR inhibitor (rapamycin), and engineered HUMSC-Exos were employed. The results showed that TGF-β1 and mTOR signaling were involved in the beneficial effects of HUMSC-Exos on tendon regeneration.

**Conclusion:**

The findings in our study suggest that PTEN/mTOR/TGF-β1 signaling cascades may be a potential pathway for HUMSC-Exos to deliver miR-29a-3p for tendon healing and implicate a novel therapeutic strategy for tendon regeneration via engineered stem cell-derived exosomes.

**Graphic abstract:**

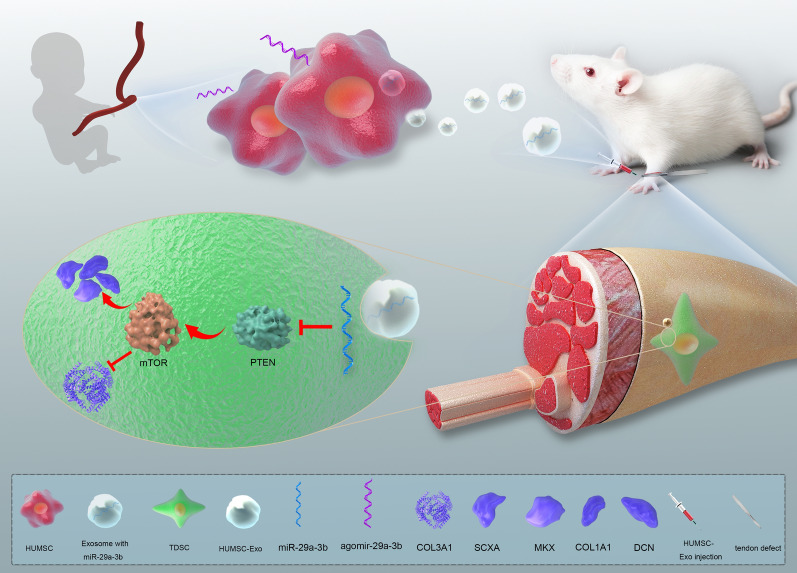

## Introduction

Tendon injuries including tendon rupture are common in sports and workplaces. However, the hypocellular and hypovascular nature of tendon makes the healing a slow and inefficient process. The impaired motor function and shortened service life finally reduced the prognosis for tendon injuries due to compromised mechanical property of the poorly regenerated tendon [1, 2]. Limitations in understanding the pathogenesis of tendon development and tendon injury have hampered the advances in clinical treatments. Although conventional surgery-based therapy methods are in use at present, they require long-term recovery and bring pain and inconvenience to patients [1, 3, 4]. Therefore, alternative treatment strategies are urgently required.

Exosomes are small (diameter of ∼ 30–200 nm) single-membrane vesicles derived from intracellular bodies. A number of evidences show that exosomes exist in almost all mammalian cells [5–7]. In cellular communication, exosomes deliver effectors such as proteins, mRNAs, or microRNAs (miRNAs/miRs) to regulate the functions of recipient cells or tissues [8]. It’s known that exosomes have huge potential in tissue regeneration and effectively reduce the side effects of cell therapy [9–13]. In recent years, various cell-derived exosomes have attracted considerable attention in musculoskeletal pathologies. Currently it is known that exosomes in musculoskeletal systems alleviate osteoarthritis [14–16], rheumatoid arthritis [17] and osteoporosis [18, 19] and promote osteointegration [20, 21], skeletal muscle or ligament repair [22]. However, there are currently short of exosomes associated with tendon regeneration. Among all the cell sources, stem cell-derived exosomes are most promising concerning their effects in tissue regeneration and repair [23–25].

Human umbilical cord mesenchymal stem cells (HUMSCs) are considered to have broad application prospects. Umbilical cords represent an attractive source of cells from available MSCs for their cost-effectiveness, efficiency, feasibility, acceptability, and universality [26, 27]. In recent years, human umbilical cord mesenchymal stem cells-derived exosomes (HUMSC-Exos) have emerged in the field of tissue regeneration and repair due to its unique advantages. The currently proven roles of HUMSC-Exos included promoting skin healing [28], vaginal epithelial regeneration [29] and fracture healing [30], alleviating type-2 diabetes [31], heart failure [32], and wound inflammation [33]. Therefore, we hypothesized that HUMSC-Exos may effectively promote tendon healing.

MiRNAs are a class of noncoding small RNAs. They may regulate gene expression at the post-transcriptional or translational level [34]. Emerging research has highlighted miRNAs as important regulators of musculoskeletal diseases, although their acting mechanisms in these complex diseases remain unclear [35]. The miR-29 family has been considered a key regulator of tissue fibrosis [36]. Recently, genome-wide and genetic analyses have revealed that miR-29a plays an important role in tendon differentiation [37]. In particular, it is suggested that miRNAs may serve as key regulators of certain molecular pathways (involving Wnt, NF-κB, mTOR, TGF-β, and sirtuins) [38]. Transcriptome analysis of mouse tendons revealed that TGF-β signaling was modified to a great extent among many pathways. Furthermore, other studies showed that the transforming growth factor-β1 (TGF-β1)/Smad 2/3 pathway regulated the development of limb tendons [39, 40]. It is widely believed that the mTOR pathway plays an important role in regulating musculoskeletal stem cell differentiation [41–44]. Notably, some latest researches suggests that mTOR signaling is effective in regulating tendon differentiation [45, 46].

In the present study, we demonstrated that HUMSC-Exos promoted tendon healing in vivo and in vitro. We further carried out deep RNA sequencing and found that HUMSC-Exos promoted higher expressions of endogenous miR-29a-3p in rat tendons. In addition, specific agonist targeting miR-29a-3p are used for HUMSCs to harvest engineered HUMSC-exos that overexpress miR-29a-3p. Later, we pleasantly revealed that HUMSC-Exos overexpress miR-29a-3p amplified the effect of tendon healing by regulating the phosphatase and tensin homolog (PTEN) /mTOR/TGF-β1 signaling pathway. The findings provide a novel alternative treatment strategy based on cell-free exosomes for tendon regeneration.

## Materials and methods

### Isolation and characterization of exosomes

Exosomes derived from HUMSCs were extracted by ultracentrifugation, as shown in a previous study [47]. In brief, the serum-free medium was collected from passages 3–6 of the HUMSCs. The collected medium was centrifuged at 1000×*g* for 10 min at 4 °C and then filtered with a 0.2-mm filter. This filtered medium was then ultracentrifuged at 100,000×*g* at 4 °C for 4 h, the pellet was then suspended in PBS, and re-ultracentrifuged at 100,000×*g* for 20 min. Finally, the pellet was resuspended in 50 μL PBS. The HUMSC-Exos were stored at − 80 °C, and their concentrations were measured using a bovine serum albumin (BSA) kit (Gibco, USA). Electron microscopy (Hitachi H7500, Tokyo, Japan) was employed to identify the morphology of the exosomes. Exosome particle size were determined using nanoparticle tracking analysis (NTA) and ZetaView PMX 120 (Particle Metrix, Meerbusch, Germany). The surface markers of HUMSC-Exos were determined by western blot analysis.

### Cell culture and treatment

Tendon-derived stem cells (TDSCs) were extracted according to established protocols [20]. Patellar tendon tissues were harvested from 4 to 6-week-old male SD rats, the tissues were minced and digested using collagenase type 1 (Sigma-Aldrich, MO, USA). The cell suspension was incubated with Dulbecco’s Modified Eagle Medium (DMEM) Low Glucose, 10% fetal bovine serum (FBS), 100 U/mL penicillin, and 100 μg/mL streptomycin (all from Gibco, USA), the medium was replaced every 2–3 days. The differentiation potentials of TDSCs were determined by osteogenic, adipogenic, and chondrogenic differentiation assays in vitro. The surface antigens of TDSCs were detected by flow cytometry (Beckman Coulter, CA, USA). Fourth generation cells were used for all experiments.

HUMSCs were purchased from the Shanghai Institute of Life Sciences, Chinese Academy of Sciences. The cells were cultured in α-MEM (Gibco, USA) supplemented with 5% UltraGRO™ (AventaCell, USA) and 1% penicillin and streptomycin (Gibco, USA), the medium was changed every 2–3 days. The 3–6th generation cell culture supernatant was used for the test. For in vitro assays, we collected the supernatant of serum-free medium with a count of 5 × 10^6^ HUMSCs, which corresponded to ~ 1.2 × 10 ^10^ particles of the donor, as determined by NTA. Four groups were assigned for in vitro studies: (1) the TDSCs without any treatment; (2) the TDSCs treated with supernatants in the presence of HUMSC-Exos; (3) the TDSCs treated with supernatants in the absence of HUMSC-Exos; (4) the TDSCs treated with HUMSC-Exos alone; all treatments were lasts for 72 h.

In the other part of the in vitro assay, agomir-NC-Exo, agomir-29a-Exo, 10 µM TGF-β1 inhibitor (SB-431542, Cell Signaling Technology, MA, USA), and 20 µM mTOR inhibitor (rapamycin, Cell Signaling Technology, MA, USA) were employed to treat TDSCs for 72 h.

For exploration of the role of miR-29a-3p in tenogenesis, specific agonist, antagonist and negative control targeting hsa-miR-29a-3p (100 nM, RiboBio, Guangzhou, China) were used to treat HUMSCs for 72 h, followed by the collection of supernatants and extraction of the exosomes.

### Luciferase assay

Full length of wild-type and mutant 3′UTR of PTEN mRNA were amplified using PCR and inserted into the pmiR-RB-Report™ double luciferase report vectors (Ribobio Co, Guangzhou, China). Cells were transfected with luciferase report vectors and agomir-29a or agomir-NC using Lipofectamine 2000 (Invitrogen). After 72 h, the luciferase activity was determined using the dual-luciferase reporter assay system (Promega, USA) according to the manufacturer’s instructions. Normalized luciferase activity was calculated as the relative value of Firefly luciferase activity to Renilla luciferase activity.

### Flow cytometry

The surface antigens of TDSCs were detected by flow cytometry (Beckman Coulter, CA, USA). The antibodies were incubated with TDSCs for 30 min at room temperature using standard procedures [48]. The cells were washed twice with PBS and resuspended in 500-μL PBS. Primary antibodies included anti-CD90 (Abcam, ab225), anti-CD34 (Abcam, ab81289), anti-CD44 (Abcam, ab157107), and anti-CD45 (Abcam, ab10558). The percentage of cells was analyzed using FlowJo Software (Tri Star Inc, Ashland, USA).

### Sirius red staining

Collagen content was determined by Sirius Red staining. TDSCs were randomly divided into the supernatant, supernatant without HUMSC-Exos, HUMSC-Exos, and the control groups. When TDSCs reached 80% confluence after culturing for 72 h, the cells were first fixed with 4% PFA for 10 min, and then washed thrice with PBS. The Sirius Red reagent (Hyclone, USA) was then added for 30 min at room temperature. The color was eluted with a 1-mL mixed solution of NaOH and absolute methanol at an equal ratio. The fluorescence was measured at a wavelength of 540 nm using a spectrophotometer (BioTek Instruments Inc., VT, USA).

### Western blot analysis

Tensile tissue, cells, or exosomes were lysed in radioimmunoprecipitation buffer (RIPA) as previously described to collect proteins [49]. They were separated by sodium dodecyl sulfate polyacrylamide gel electrophoresis gel (SDS-PAGE). The protein was transferred to a polyvinylidene fluoride (PVDF, Millipore, USA) membrane via an electroporation system, blocked with 5% nonfat milk and then incubated at 4 °C overnight with the following primary antibodies: anti-CD9 (Abcam, ab92726), anti-CD63 (Abcam, ab217345), anti-Alix (Abcam, ab117600), anti-COL1A1 (Abcam, ab34710), anti-COL3A1 (Abcam, ab7778), anti-decorin (DCN; Affinity Biosciences, DF6543), anti-scleraxis (SCXA; Abcam, ab58655), anti-tenomodulin (TNMD; Abcam, ab203676), anti-mohawk (MKX; Santa Cruz Biotechnology, sc-515878), anti-TGF-β1 (Abcam, ab92486), anti-p-Smad3 (Abcam, ab52903), anti-p-mTOR (Cell Signaling Technology, #5536). Anti-β-actin (Abcam, ab8226) was used as control. The Bands were scanned using an enhanced chemiluminescence detection system and quantified using ImageJ software.

### Quantitative RT-PCR analysis (Q-RT-PCR)

For miRNA detection, miRNA was extracted using a miRNA Isolation Kit (BioFlux, Japan), and then, PCR reactions were performed, U6 was used as the standardized internal reference.

The primer sequences used for the analysis were as follows:

- hsa-miR-29a-3p: forward 5′-CTCAACTGGTGTCGTGGAGTCGGCAATTCAGTTGAGTAACCGAT-3′, reverse 5′-ACACTCCAGCTGGGTAGCACCATCTGAAAT-3′
- rno-miR-29a-3p: forward 5′-CTCAACTGGTGTCGTGGAGTCGGCAATTCAGTTGAGTAACCGAT-3′, reverse 5′-ACACTCCAGCTGGGTAGCACCATCTGAAAT-3′
- U6 primers: forward 5′-CTCGCTTCGGCAGCACA-3′, reverse 5′-AACGCTTCACGAATTTGCGT-3′.

### Exosomal repair of rat tendon defects in vivo

Animal procedures in this study were approved by the Animal Care Committee of Shanghai Jiao Tong University Affiliated Sixth People’s Hospital (No. DWLL2019-0290). Adult male Sprague–Dawley (SD) rats (weight: 200–220 g) were randomly assigned to different treatment groups (n = 30 per group). The animals were anesthetized with a 3% pentobarbital intraperitoneal injection, and then, a 5 × 1-mm^2^ rectangular full-thickness defect was introduced to the left Achilles tendon of the rats. In the first part of the animal experiment, rats subjected to the surgical operation were randomly divided into three groups: (1) sham group: no other treatment after surgical procedures, (2) fibrin group: implantation with 50 μL fibrin glue at the tendon defect site, and (3) HUMSC-Exo group: implantation with 50 μL fibrin glue + 100 μg HUMSC-Exos at the tendon defect site. The regenerated tendons were collected at 2 and 4 weeks for histology, immunochemistry, and biomechanical analysis.

In the second part of the animal experiment, the rats subjected to the surgical operation were randomly allocated into three groups: (1) sham group: no other treatment after surgical procedures, (2) Agomir-NC-Exo group: implantation with 50 μL fibrin glue + 100 μg agomir-29a-Exo at the tendon defect site, and (3) Agomir-29a-Exo group: implantation with 50 μL fibrin glue + 100 μg agomir-29a-Exo at the tendon defect site. The treated tendons were collected at 4 weeks for immunochemistry and biomechanical evaluation.

### Small RNA sequencing

The total RNAs of the Achilles tendons were extracted and used for miRNA sequencing (miRNA-seq). Library preparation and miRNA-seq were performed by Shanghai Jiao Tong University. After fractionating the total RNA from the tendon tissues, small RNAs ranging from 18 to 30 nucleotides (nt) were used for library preparation. The PCR products were sequenced using the HiSeq 2500 platform (Illumina, San Diego, CA, USA).

### Histological staining and evaluation

Rats were euthanized at 2 and 4 weeks to collect tendon tissues. The tissues were fixed in 10% buffered formalin for 48 h to obtain paraffin sections (5 μm). The paraffin sections were processed for hematoxylin and eosin (H&E) staining or Masson trichrome staining and were observed under an optical microscope (Leica Microsystems, Wetzlar, Germany). Semiquantitative analysis was conducted as published previously (n = 6 per group) [24].

### Immunohistochemistry and cellular immunofluorescence

Tendon sections were stained using a standardized procedure and incubated overnight at 4 °C with primary antibodies: anti-COL1A1 (Abcam, ab34710), anti-SCXA (Abcam, ab58655), and anti-TNMD (Abcam, ab203676). The sections were washed three times with PBS the next day and incubated with the secondary antibodies for 30 min at room temperature. 4′,6-Dimercapto-2-phenylindole (DAPI, Gibco, USA) was used for nuclear staining.

For cellular immunofluorescence (IF), cell slides were fixed with 4% paraformaldehyde (PFA) and incubated overnight at 4 °C with primary antibodies: anti-COL1A1 (Abcam, ab34710), anti-COL3A1 (Abcam, ab7778), anti-DCN (Affinity Biosciences, DF6543), and anti-TNMD (Abcam, ab203676). The slides were incubated with fluorescently conjugated secondary antibodies for 30 min at room temperature in the dark. DAPI was used for nuclear staining. The image was observed and captured with a fluorescence microscope (Olympus, Tokyo, Japan). ImageJ software was used for semi-quantitative analysis.

### Biomechanical test

Achilles tendons were collected at 2 and 4 weeks (n = 6 per group). The tendon tissues were preserved in a moist gauze to prevent drying. The proximal and distal ends of the tendon were respectively attached to the special fixture. The proximal end was first frozen with liquid nitrogen and was fixed with a clamp [23]. All tendons were mounted on the Instron 5569 Universal Testing System (Instron, MA, USA). The tendons were subjected to a tensile test (axial velocity: 30 mm/min, with 0.1-N preload using a 100-N load cell) until maximum load failure was achieved. The biomechanical properties of the Achilles tendon were evaluated by ultimate tensile strength (UTS) (N), stiffness (N/mm), and Young’s modulus.

### Statistical analysis

All data are presented as mean ± standard deviation (SD). The comparisons of data between multiple groups were analyzed using one-way analysis of variance (ANOVA) and those between two groups were analyzed using Student’s *t-*test to assess the significance of differences. All data analyses were performed with Prism 7.0 (GraphPad). The level of significance was set at *P* value < 0.05.

## Results

### Identification and characterization of HUMSC-Exos and TDSCs

**Fig. 1:**
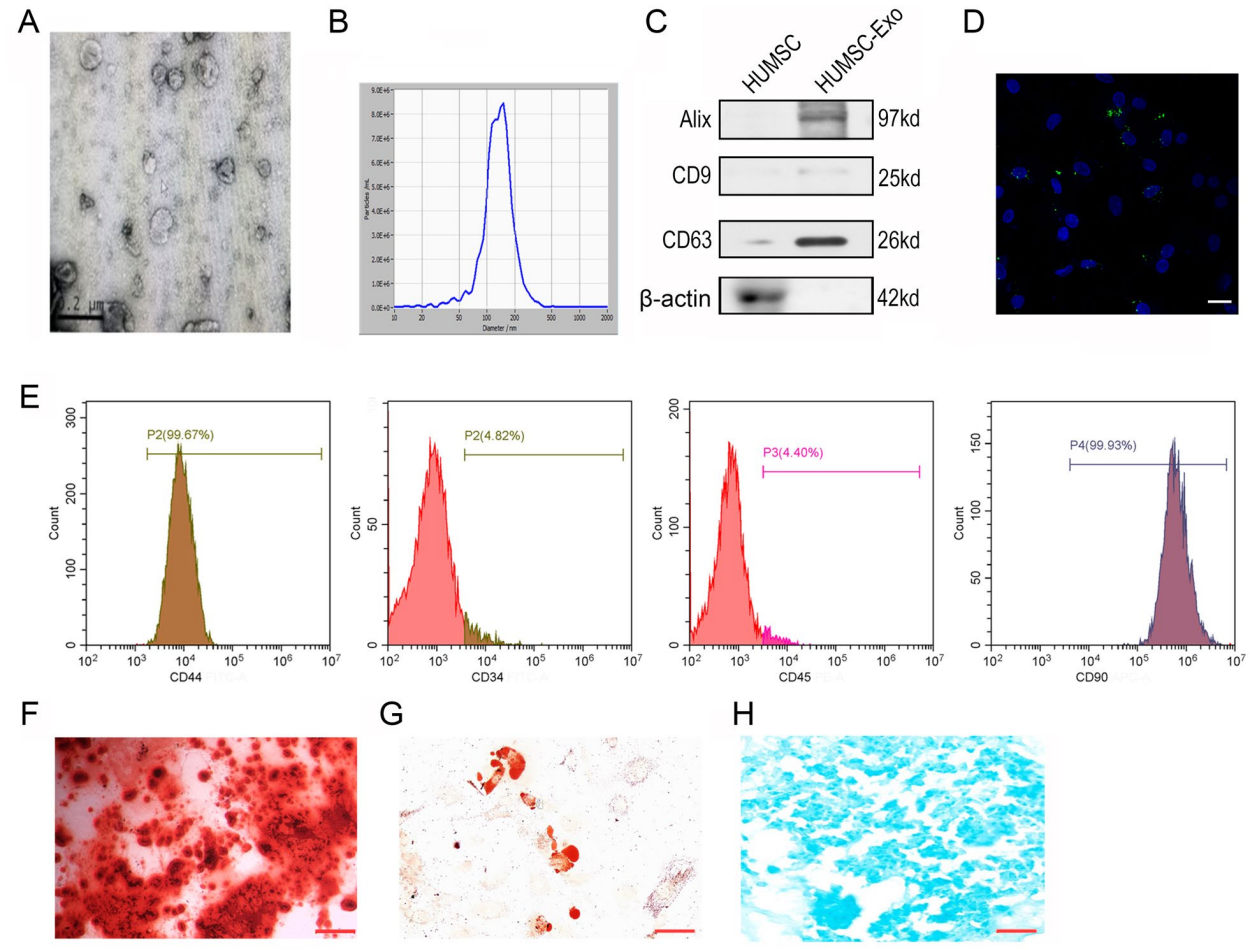
Identification of exosomes and TDSCs. **A** Representative image of HUMSC-Exos observed by electron microscopy. Scale bar: 200 nm. **B** NTA observation of the size of the HUMSC-Exos. **C** Surface markers of HUMSCs and HUMSC-Exos measured using western blot. **D** Cellular uptake of HUMSC-Exos by TDSCs observed using PKH67 staining, scale bar: 20 μm. **E** Flow cytometry analysis of the surface antigens of TDSCs (n = 5 per group). TDSCs could differentiate into osteoblasts, adipocytes, and chondrocytes. **F** Alizarin Red S staining, scale bar: 50 μm. **G** Oil Red O staining, scale bar: 25 μm. **H** Alcian Blue staining, scale bar: 25 μm

As shown in Fig. 1, an electron microscope was employed to observe the morphology of the exosomes (Fig. 1A). The NTA indicated an abundance of HUMSC-derived vesicles with an average diameter of 137.6 nm (Fig. 1B), further, the western blot analysis showed that exosomal specific markers including CD9, CD63, and Alix were positive (Fig. 1C). The above results indicated their identity as exosomes. Additionally, PKH67 staining of Exos for cellular uptake observation showed that HUMSC-Exos were effectively absorbed by TDSCs (Fig. 1D).

To identify whether cells cultured using low-density plating were TDSCs, we examined the multipotential differentiation of cells. Osteogenic differentiation showed that most cells had mineralized calcium deposits (Fig. 1F) and Oil Red O staining results confirmed adipogenic differentiation (Fig. 1G). The ability of the cultured cells to differentiate into chondrocytes was demonstrated by Alcian Blue staining (Fig. 1H). In addition, we employed flow cytometry to identify the surface antigens of TDSCs. The results showed that CD34 and CD45 were negatively expressed, while CD90 and CD44 had highly positive expressions (Fig. 1E). The above results indicated that the cells isolated from the rat patellar tendons were TDSCs.

### HUMSC-Exos promoted the expression of tendon markers in TDSCs in vitro

**Fig. 2:**
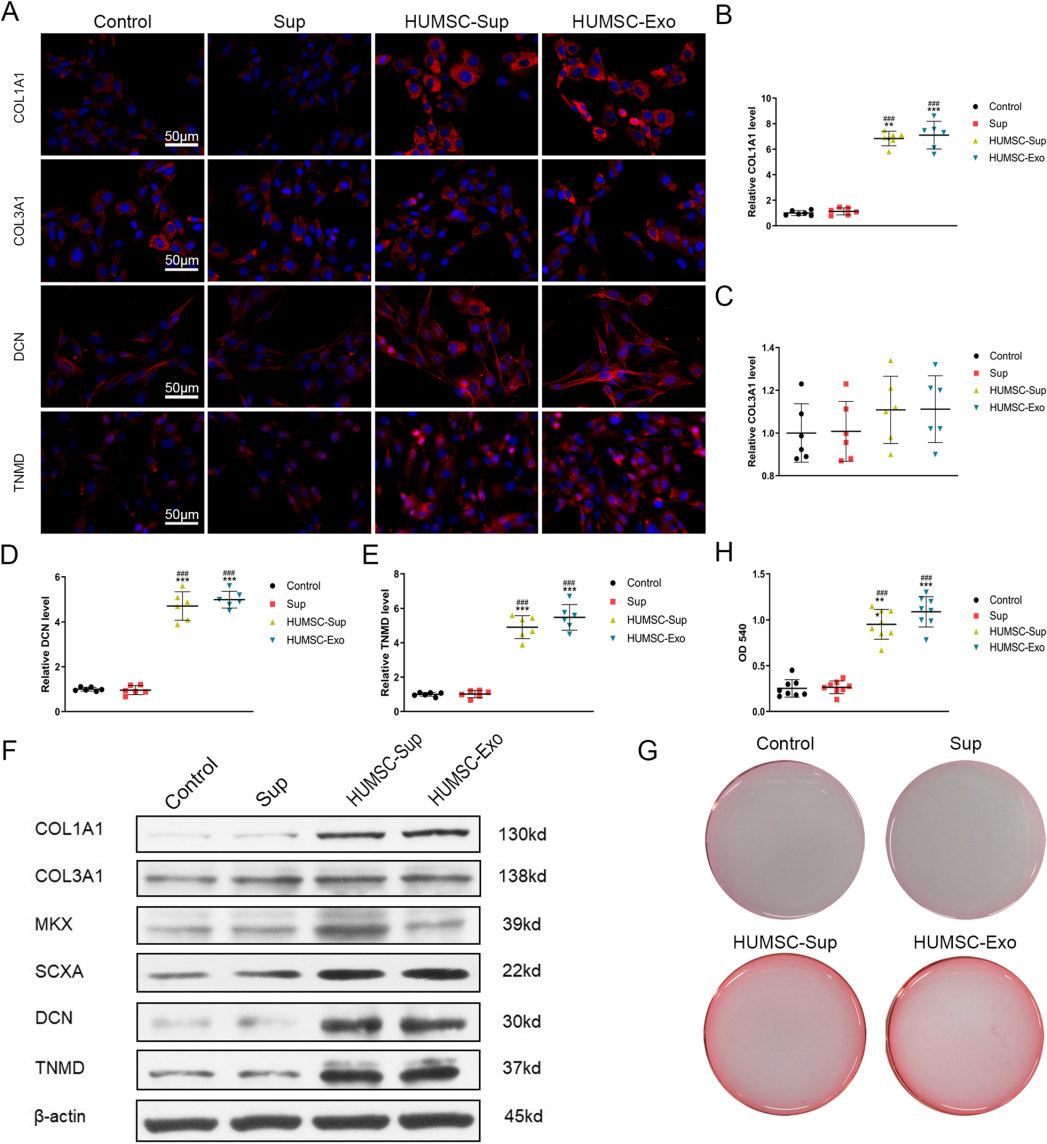
HUMSC-Exos promoted expression of tendon markers. **A** Representative cellular immunofluorescence images of COL1A1, COL3A1, DCN, TNMD (red), and the nuclei stained with DAPI (blue), scale bar: 50 μm. **B**–**E** Quantification of cellular immunofluorescence (n = 6 per group). *P < 0.05 vs. control. ^*#*^*P* < 0.05 vs. Sup group. **F** Representative western blot images of COL1A1, COL3A1, MKX, SCXA, DCN, and TNMD. **G**, **H**. Sirius Red staining for collagen deposition (n = 6 per group). ***P < 0.001 vs. control group. ^*###*^*P* < 0.001 vs. Sup group

To determine the effect of HUMSC-Exos on TDSCs, a serum-free medium supernatant with a count of 5 × 10^6^ HUMSCs was collected, which corresponds to ~ 1.2 × 10^10^ particles of the donor, as determined by NTA. TDSCs were treated with supernatants in the presence or absence of HUMSC-Exos or with HUMSC-Exos alone for 72 h. HUMSC-Exos and HUMSC supernatant-containing exosomes (HUMSC-Sup) promoted the expression of tendon-specific markers such as COL1A1, SCXA, DCN, and TNMD compared with the control group, and there was no statistical difference between the previous two groups. However, COL3A1 expression did not show any significant difference among the four groups. The level of MKX significantly increased in the supernatant group compared with that in the control group, however, no increase was observed in the HUMSC-Exo group. For all indicators, the HUMSC-Exo group obtained significantly better results than the supernatant without exosomes (Sup) group except for COL3A1 (Fig. 2F). The results of IF also reached similar conclusions. Specifically, the staining density of COL1A1 in HUMSC-Sup or HUMSC-Exo group was both much stronger than that in the Control or Sup group, though no significant difference was found between the HUMSC-Sup and HUMSC-Exo group (Fig. 2A, B). The same is true for DCN and TNMD staining (Fig. 2A, D, E), while the staining density of COL3A1 was similar among groups (Fig. 2A, C). We next performed Sirius Red staining to assess the collagen expression of TDSCs. The results showed that HUMSC-Exos significantly increased collagen deposition (Fig. 2G, H). All the above results indicated that HUMSC-Exos significantly promoted the differentiation of TDSCs into the tendon and accelerated the expression of tendon-specific genes.

### HUMSC-Exos accelerated tendon healing in vivo

**Fig. 3:**
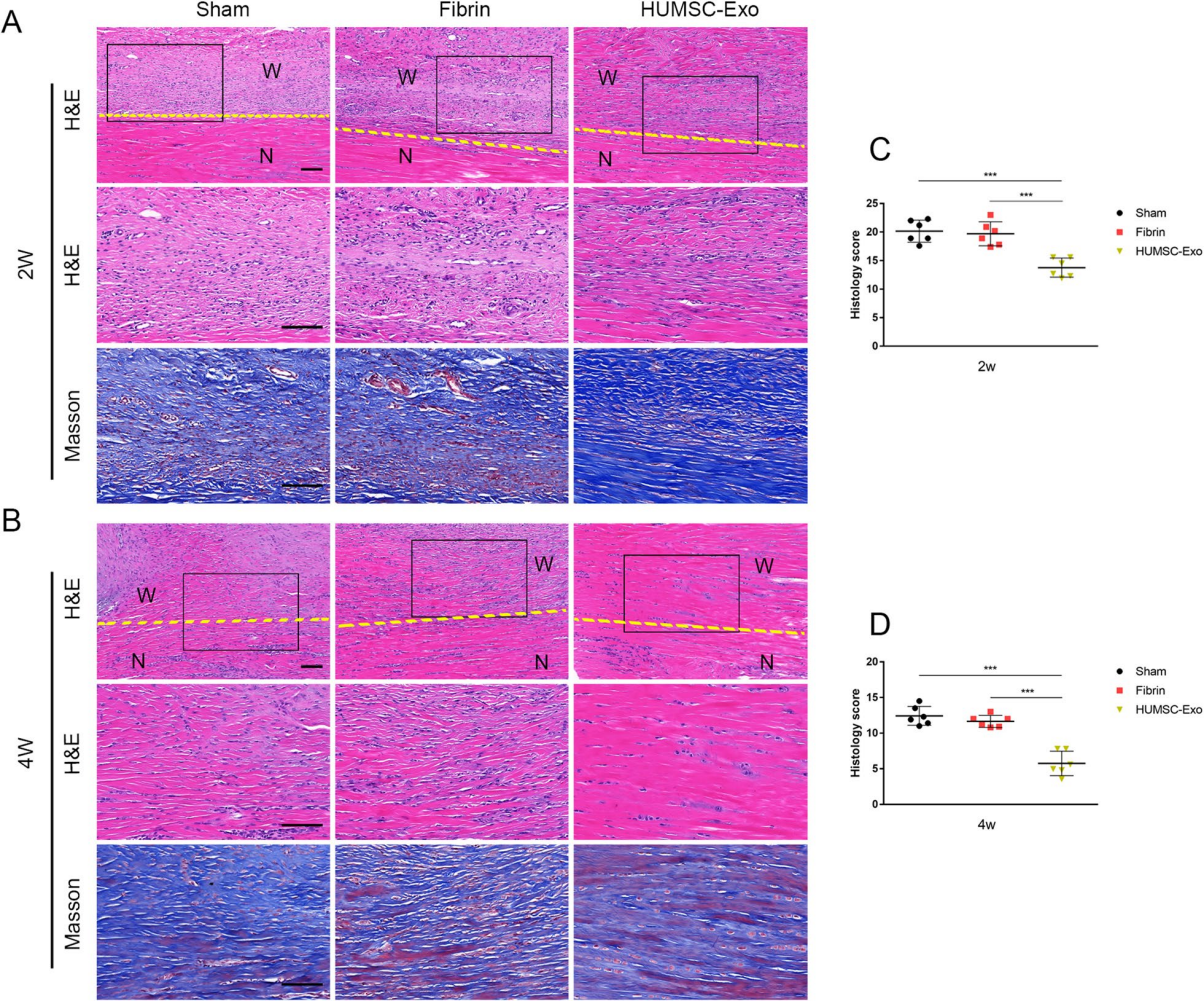
Histological examination after application of HUMSC-Exos at injured tendons. **A**, **B** Representative H&E staining and Masson staining. **C**, **D** Histology score of tendons at 2 and 4 weeks after injury. HUMSC-Exo-treated tendons showed better tendon morphology and density than the sham and fibrin groups. Histological evaluation, the HUMSC-Exo group scores were lower than those of the sham group (n = 6 per group). Scale bar: 100 μm. W: wound region, T: normal region. The yellow dotted line indicates the boundary between the new tendon tissue and normal tissue after injury. ***P < 0.001

We validated the effect of HUMSC-Exos in a rat tendon repair model. The HUMSC-Exos were transplanted at the site of the tendon defect with fibrin glue as the carrier, and the rat tendon specimens were collected at 2 and 4 weeks, respectively. Histological analysis showed that HUMSC-Exo-treated tendons exhibited more regular and dense connective tissue filling. Masson staining showed that the collagen deposition in the HUMSC-Exo group was significantly higher than that in the sham and fibrin groups (Fig. 3A, B). The histological scores of the HUMSC-Exo group were significantly lower than those of the sham and fibrin groups. The results were similar at 2 and 4 weeks postoperatively (2 weeks: 14.1 ± 1.7 vs. 20.8 ± 1.7, *P* < 0.05, 4 weeks: 5.9 ± 1.9 vs. 12.5 ± 1.5, *P* < 0.05) (Fig. 3C, D). Cells in the HUMSC-Exo-treated group were distributed in a spindle-like arrangement and the collagen fibers were dense and regular, while for the other two control groups, they were loose and relatively sparse.

**Fig. 4:**
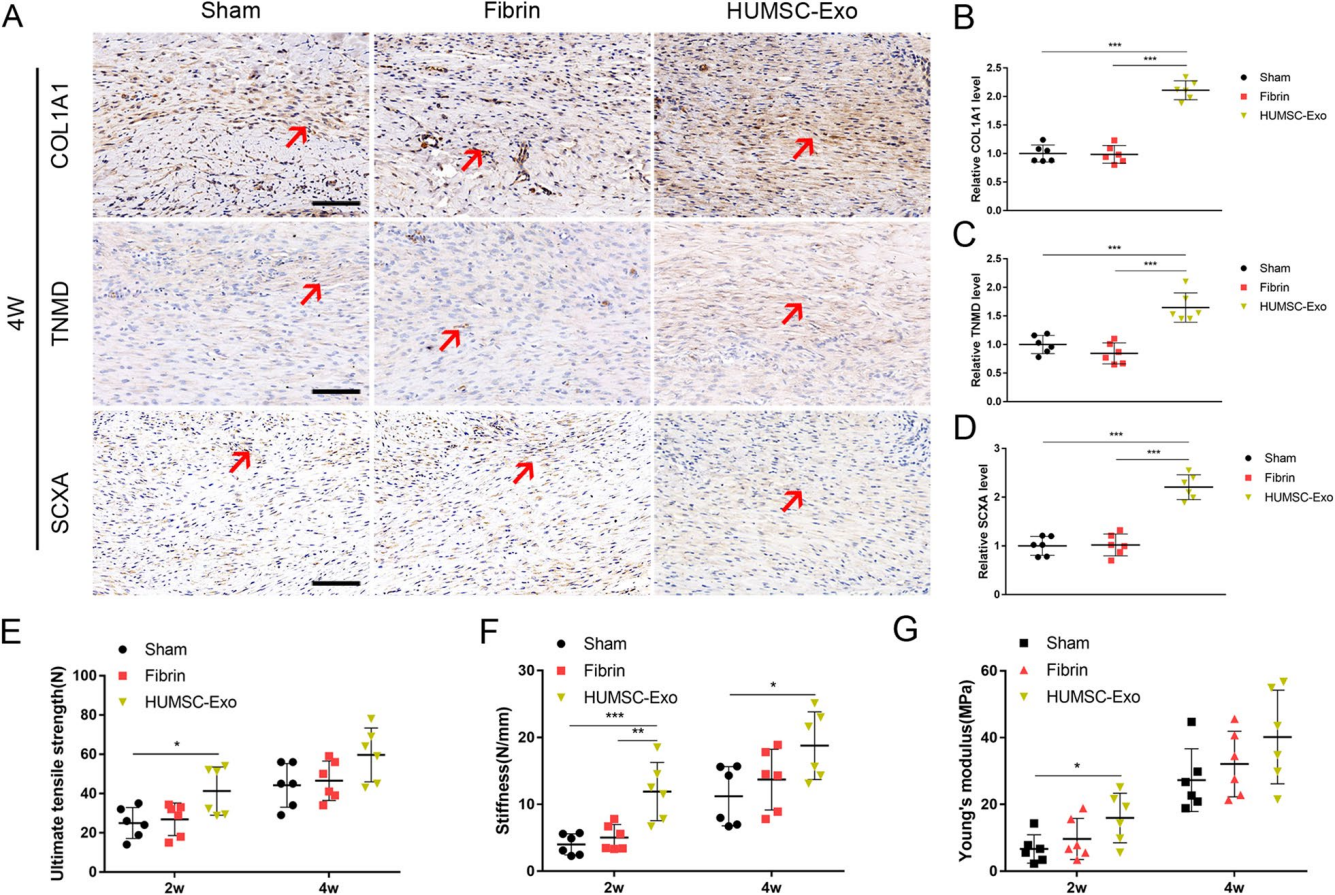
HUMSC-Exos improved histochemical and biomechanical performance in vivo*.* **A**–**D** Representative immunohistochemical staining and quantification of COL1A1, TNMD, and SCXA (n = 6 per group). Scale bar: 100 μm. Red arrow: tendon marker (COL1A1, TNMD, and SCXA) positive areas. ***P < 0.001. **E**–**G** Biomechanical analysis of ultimate tensile strength (N), stiffness (N/mm), and Young’s modulus (MPa) (n = 6 per group). *P < 0.05, **P < 0.01, ***P < 0.001

**Fig. 5:**
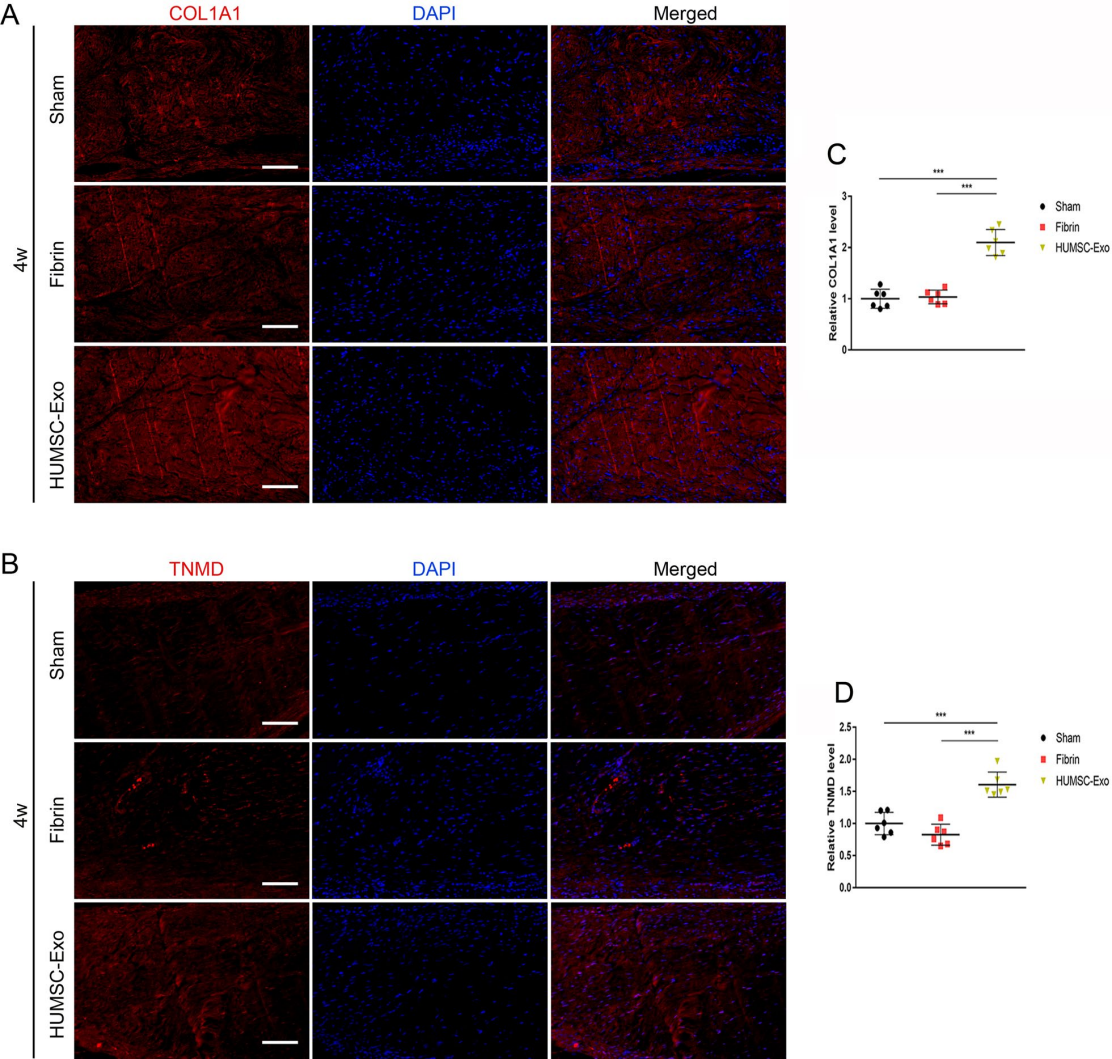
HUMSC-Exos improved the performance of immunostaining of tendon regeneration in vivo. **A** Representative immunostaining staining of COL1A1. **B** Representative immunostaining staining of TNMD. **C** Quantification of COL1A1 staining. **D** Quantification of TNMD staining (n = 6 per group). Scale bar: 100 μm. ***P < 0.001

We evaluated the expression of tendon markers by immunohistochemistry at 4 weeks after surgery. The results showed that the relative expressions of tendon markers COL1A1, TNMD, and SCXA in the HUMSC-Exo group were 2.1-fold, 1.6-fold, and 2.2-fold higher than that of the sham group, respectively (Fig. 4A–D). In addition, we further performed immunofluorescence staining of COL1A1 and TNMD. The results were similar with those of immunohistochemical staining (Fig. 5A–D). These results indicated that HUMSC-Exos significantly promoted the deposition of the extracellular matrix (ECM) of the tendon and accelerated tendon healing.

Next, we examined the biomechanical properties of the healing tendons. During the test, five samples were discarded because of slippage (2 from the HUMSC-Exo group, 2 from the fibrin group, and 1 from the sham group), and a total of 36 samples were tested (n = 6 per group). The biomechanical properties of the tendons in the HUMSC-Exo group were superior to those in the sham group. In particular, in the HUMSC-Exo group, the UTS was significantly higher than that in the sham group at 2 weeks (41.3 ± 12.3 N vs. 25.0 ± 7.9 N, *P* < 0.05), however, there was no statistical difference between the HUMSC-Exo and the fibrin groups (41.3 ± 12.3 N vs. 26.9 ± 8.3 N, *P* > 0.05). At 4 weeks, there was no statistical significance among three groups in terms of UTS, however, the highest tensile strength was obtained in the HUMSC-Exo group (59.7 ± 13.7 N vs. 46.5 ± 10.0 N vs. 44.2 ± 11.0 N, *P* > 0.05) (Fig. 4E). In terms of stiffness, the HUMSC-Exo group was significantly better than the sham group at 2 and 4 weeks, respectively (12.1 ± 3.5 N/mm vs. 4.5 ± 2.1 N/mm, *P* < 0.05 and 18.5 ± 5.5 N/mm vs. 11.1 ± 4.8 N/mm, *P* < 0.05). The HUMSC-Exo group was superior to the fibrin group at 2 weeks (12.1 ± 3.5 N/mm vs. 5.0 ± 2.0 N/mm, *P* < 0.05), however, there was no statistically significance at 4 weeks (18.5 ± 5.5 N/mm vs. 13.7 ± 4.5 N/mm, *P* > 0.05) (Fig. 4F). The Young’s modulus of the HUMSC-Exo group was higher than that of the sham group at 2 weeks (16.0 ± 7.4 MPa vs. 6.7 ± 4.3 MPa, *P* < 0.05), but there was no significant difference at 4 weeks (40.2 ± 14.0 MPa vs. 29.3 ± 14.0 MPa, *P* > 0.05) (Fig. 4G). All of the above data indicated that HUMSC-Exos enhanced biomechanical properties.

### MiRNA sequencing showed a significant increase in miR-29a-3p expression in HUMSC-Exo-treated tendons

**Fig. 6:**
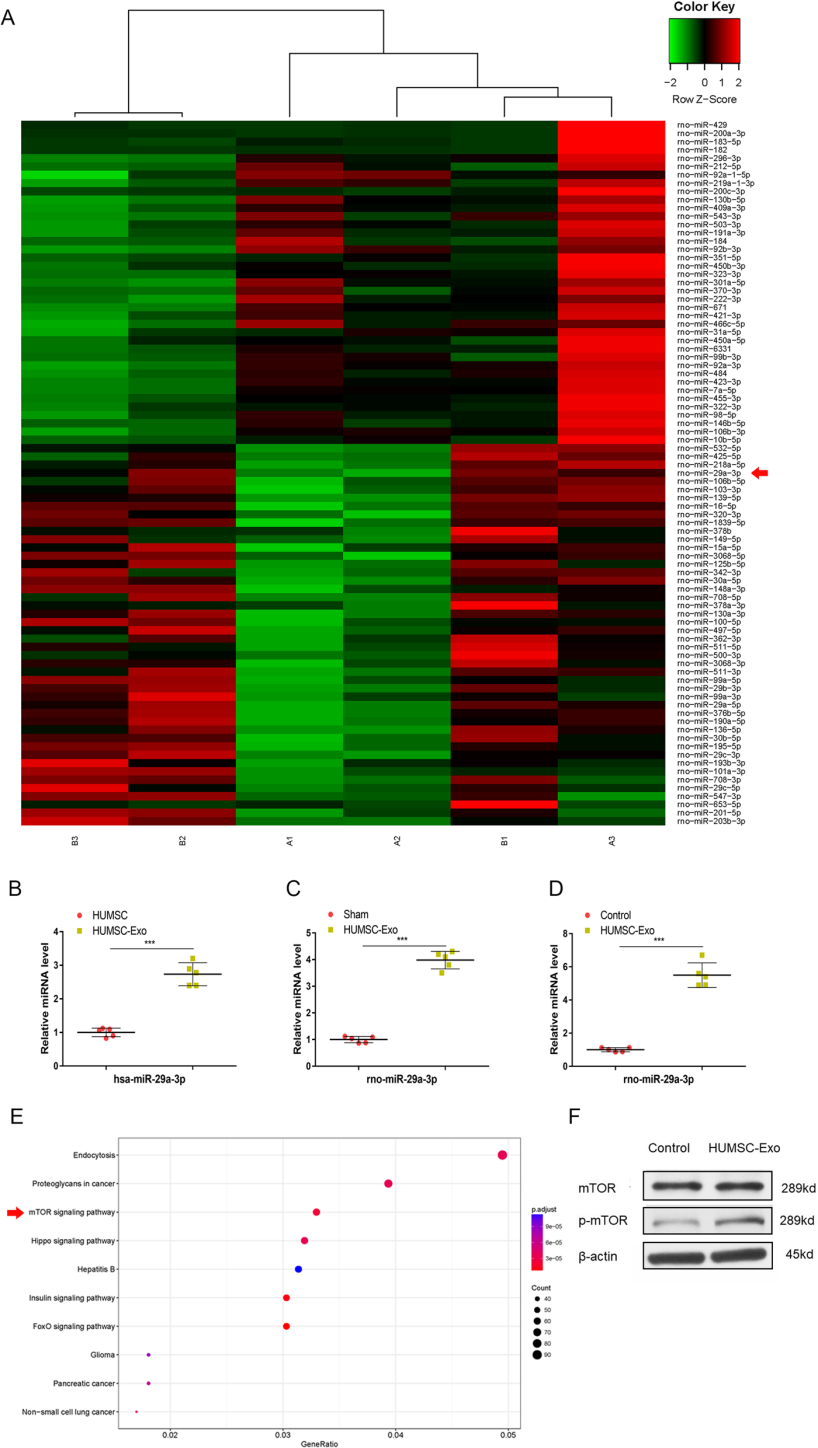
MiRNA sequencing analysis. **A** Heat map of miRNA sequencing (miRNA-seq) analysis of rat tendons treated with or without HUMSC-Exos (red: high expression, green: low expression, **A1**–**A3** sham group, **B1**–**B3** HUMSC-Exo group (n = 3 per group), and red arrow: rno-miR-29a-3p. **B**, **C**, **D** The expression of hsa-miR-29a-3p and rno-miR-29a-3p determined by Q-RT-PCR (n = 5 per group). ***P < 0.001. **E** KEGG analysis chart of miRNA-seq. **F** Representative western blot image of p-mTOR and mTOR

To investigate the detailed mechanism of HUMSC-Exos accelerating tendon repair, we performed high-throughput miRNA-seq of tendon tissues treated with or without HUMSC-Exos, wherein 85 of the known miRNAs were differentially expressed, with miR-29a-3p being one of the most distinctly differentially expressed miRNAs (Fig. 6A). Next, we examined the expression of miR-29a-3p between HUMSCs and HUMSC-Exos as well as among differently treated tendons using Q-RT-PCR. The results showed that the level of hsa-miR-29a-3p was significantly higher in HUMSC-Exos than that in HUMSCs (Fig. 6B), and rno-miR-29a-3p expression was also significantly elevated in the HUMSC-Exo-treated tendon compared with that in the sham group (Fig. 6C). For HUMSC-Exo-treated TDSCs, the expression of rno-miR-29a-3p was also significantly enhanced compared with that in the control group (Fig. 6D). Interestingly, we found the hsa-miR-29a-3p and rno-miR-29a-3p shared the same sequence, thus the upregulated rno-miR-29a-3p presence in HUMSC-Exo-treated tendon is likely directly granted by HUMSC-Exos.

In recent years, the miR-29 family has attracted increasing attention for its role in tendinopathy and tendon development. Our miRNA-seq results also suggest a potential role for miR-29a-3p in tendon healing, though the downstream mechanism yet to be determined. However, in view of the results of the KEGG analysis of bioinformatics, as shown in Fig. 6E, the mTOR signaling pathway are highly enriched and thus became a candidate target for our focus. Accordingly, WB results confirmed an increase in p-mTOR expression in HUMSC-Exo-treated tendons (Fig. 6F), indicating the activation of mTOR signaling. Therefore, we hypothesized that HUMSC may promote tendon healing by delivering exosomal miR-29a-3p to regulate the mTOR signaling pathway.

### MiR-29a-3p targeted PTEN to mediate the effects of HUMSC-Exos on mTOR signaling

**Fig. 7:**
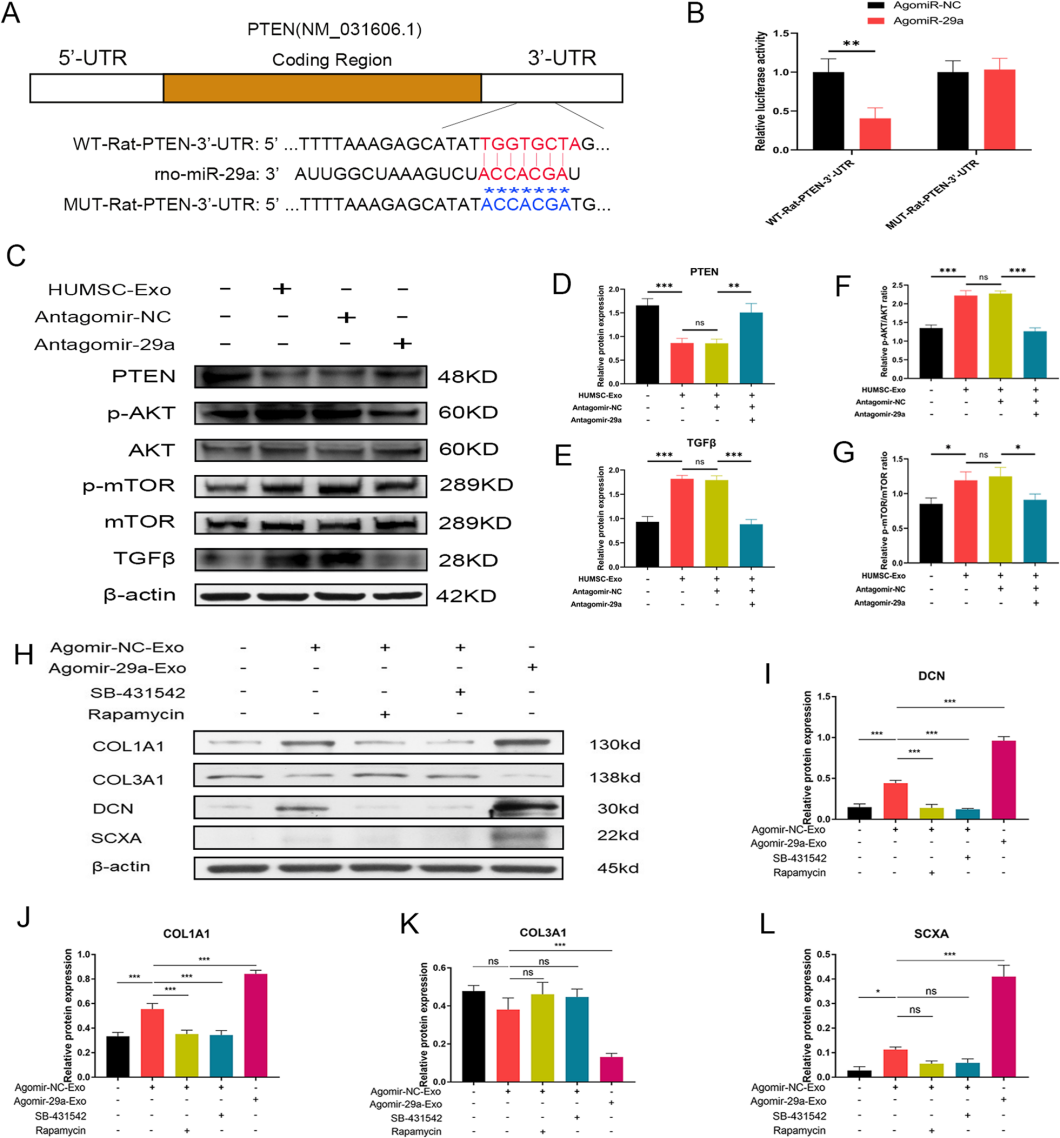
HUMSC-Exos activated PTEN/mTOR/TGF-β1 signaling to promote tendon repair by delivering miR-29a-3p. **A** Base sequence of hsa-miR-29a-3p and illustration of luciferase reporter gene containing the wild-type and mutant 3′UTR region of PTEN with binding sequence of hsa-miR-29a-3p. **B** Relative luciferase activity after transfection. **C** Representative western blot images of PTEN, TGF-β1, p-AKT, AKT, p-mTOR and mTOR. **D**–**G** Quantification of the relative gray level of the PTEN, TGF-β1 and relative ratio of p-AKT/AKT, p-mTOR/mTOR. **H** Representative western blot images of COL1A1, COL3A1, DCN, and SCXA. **I**–**L** Quantitative analysis of COL1A1, COL3A1, DCN, and SCXA (n = 3 per group). *P < 0.05, **P < 0.01, ***P < 0.001. *ns* not significant

In order to find the direct binding target of miR-29a-3p that might modulate the mTOR signaling pathway, we used the Targetscan algorithm to predict the binding domain of miR-29a-3p and found PTEN as a potential candidate. To confirm this, we constructed luciferase vectors containing the PTEN 3′UTR sequence or mutant PTEN 3′UTR sequence and transfected the TDSCs. After treated with the miR-29a-3p agonist, we could observe that the luciferase activity of the wild-type vector was significantly inhibited while the luciferase activity of the mutant vector remained unchanged, successfully verifying the direct interaction between PTEN and miR-29a-3p (Fig. 7A, B). In view of this finding, we next determined whether the PTEN bridged the miR-29a-3p containing HUMSC-Exos and the mTOR signaling. As expected, HUMSC-Exos effectively downregulated the PTEN expression in TDSC. However, administration of miR-29a-3p inhibitor significantly rescued the PTEN expression, indicating the miR-29a-3p dependence of above effects by HUMSC-Exos (Fig. 7C, D). Accordingly, downstream of PTEN, the AKT signaling was dramatically activated by the HUMSC-Exos, manifested by the elevated phosphorylation of AKT protein, which accounted for the upregulated mTOR phosphorylation (Fig. 7C, F, G). Likewise, these effects were also prominently reversed by the miR-29a-3p inhibitor. Moreover, as an important effector of mTOR signaling and anabolic cytokine, TGF-β1 synthesis could be efficiently promoted by the HUMSC-Exos but blocked by miR-29a-3p (Fig. 7C, E). To sum up, it could be postulated that miR-29a-3p mediated the promoting effects of HUMSC-Exos on mTOR signaling through directly targeting PTEN.

### HUMSC-Exos activated PTEN/mTOR/TGF-β1 signaling via exosomal miR-29a-3p to promote the expression of tendon markers in vitro

Previous evidence supported that TGF-β1 was one of the classical effectors downstream of the mTOR pathway. We found that TGF-β1 was significantly upregulated after HUMSC-Exo treatment but downregulated in the miR-29a-3p agonist engineered group, along with the similar changes in mTOR signaling, suggesting an underlying correlation between the mTOR/TGF-β1 pathway and miR-29a-3p. We next validated our conjecture that the mTOR/TGF-β1 pathway caused the beneficial effects of HUMSC-Exo on tendon markers expression of TDSCs. We treated TDSCs with different combinations of TGF-β1 inhibitor (SB-431542), mTOR inhibitor (rapamycin), agomir-29a and agomir-NC engineered HUMSC-Exos for 72 h. WB results showed that the agomir-29a significantly enhanced the effect of HUMSC-Exos on the expression of tendon markers including COL1A1, COL3A1, DCN, and SCXA. Rapamycin partially reversed the gain effect of HUMSC-Exos on the tendon markers. The TGF-β1 inhibitor suppressed the gain effect of HUMSC-Exos on the tendon markers as well (Fig. 7H–L). The above results indicated that HUMSC-Exos regulated the PTEN/mTOR/TGF-β1 pathway to promote tendon healing by delivering miR-29a-3p.

### MiR-29a-3p agonist amplified HUMSC-Exos’ effect on improving tendon healing in vivo

**Fig. 8:**
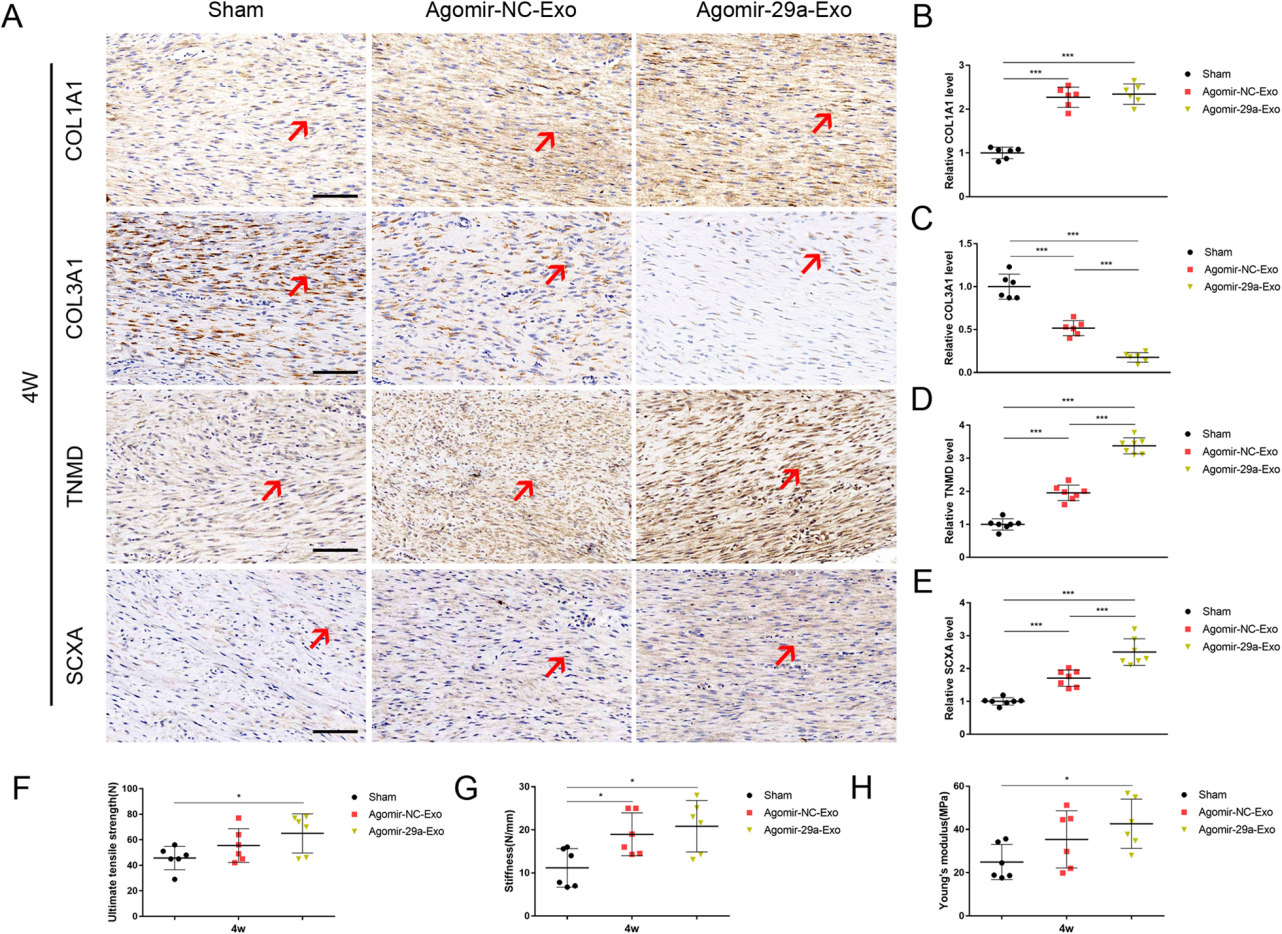
Agomir-29a-Exo amplified gain effect on improving tendon healing. **A** Representative immunohistochemical staining of COL1A1, COL3A1, TNMD, and SCXA. Quantification of the **B** COL1A1, **C** COL3A1, **D** TNMD, and **E** SCXA staining (n = 6 per group, scale bar: 100 μm). Red arrow: tendon marker (COL1A1, COL3A1, TNMD, and SCXA) positive areas. ***P < 0.001. Biomechanical analysis of **F** ultimate tensile strength (N), **G** stiffness (N/mm), and **H** Young’s modulus (MPa) (n = 6 per group). *P < 0.05

Based on abovementioned findings, to test whether miR-29a-3p agonist could further enhanced the HUMSC-Exos’ effect for tendon healing in vivo, a specific agonist and negative control targeting hsa-miR-29a-3p was employed to treat HUMSCs for 72 h, and then extracted agomir-NC-Exo and agomir-29a-Exo, respectively. At 4 weeks, we collected rat tendon specimens for immunohistochemistry. The results showed that agomir-29a-Exo significantly increased the expression of the tendon markers, TNMD and SCXA (Fig. 8A, D, E). Further, agomir-29a-Exo significantly elevated the expression of COL1A1 compared with that in the sham group, however, there was no statistical difference between the agomir-29a-Exo group and agomir-NC-Exo group (Fig. 8A, B). In particular, agomir-29a-Exo significantly reduced the expression of COL3A1, and agomir-29a-Exo decreased it further (Fig. 8A, C). These findings essentially indicate that miR-29a-3p-agonist-treated HUMSC-Exos further promote the expression of tendon markers compared with normal HUMSC-Exos.

**Fig. 9:**
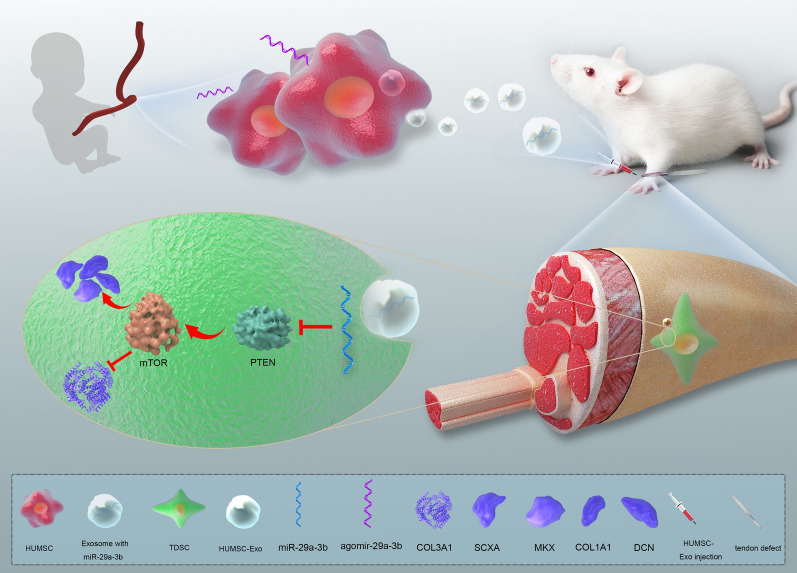
The underlying mechanism of HUMSC-Exos dependent tendon repair

The biomechanical data showed that the agomir-29a-Exo-treated tendons had higher average scores in terms of UTS, stiffness, and Young’s modulus than agomir-NC-Exo-treated tendons, however, none of the properties were significantly different between the two groups (Fig. 8F–H). This may be due to the relatively limited sample size (n = 6). Further, all mechanical properties of agomir-29a-Exo-treated tendons were significantly improved compared with those in the sham group. These findings suggested that agomir-29a-Exo-Exos further promoted tendon healing in vivo and miR-29a-3p may be a potential mediator for optimized exosomes. A proposed underlying mechanism of HUMSC-Exos in promotion of tendon repair was presented as Fig. 9.

## Discussion

In this study, we demonstrated that HUMSC-Exos might promote the expression of tendon markers and increase collagen secretion in TDSCs. In vivo, we found that HUMSC-Exos accelerated the tendon healing process and demonstrated excellent biomechanical properties. The miRNA-seq analysis showed that HUMSC-Exos promoted higher expressions of endogenous miR-29a-3p in rat tendons. Further, specific agonist targeting miR-29a-3p were employed to modify HUMSC-Exos to obtain engineered exosomes that overexpress miR-29a-3p. Subsequent in vivo and in vitro assays showed that HUMSC-Exos overexpressing miR-29a-3p further amplified the gain effect on tendon healing, and it might be accomplished by manipulating the PTEN/mTOR/TGF-β1 pathway. Our study suggested that HUMSCs may promote tendon healing by delivering exosomal miR-29a-3p to regulate the PTEN/mTOR/TGF-β1 pathway, thus providing a new therapeutic potential for tendon healing.

Stem cell transplantation from different sources such as embryos, fats, and tendons combined with tissue engineering has led to the rise of stem cell therapies for tendon injuries or tendinopathy [50]. In fact, cell-free exosome therapy is emerging as a new generation of research focus and is increasingly being used in tissue regeneration because of its unique immune exemption, non-tumorigenicity, high stability, and ease of engineering modification [51, 52]. However, there are few reports on stem cell-derived exosomes in tendon injury healing. In this study, we demonstrated that HUMSC-derived exosomes promoted tendon healing in vivo and in vitro. The results of this study would act as footnotes to the potential of HUMSC-Exos in the field of tissue regeneration and injury repair, while providing a novel therapeutic strategy for tendon regeneration.

Recent studies have emphasized that miRNAs determine the ECM composition by regulating leukocyte function and stromal cell differentiation [35, 53]. It is well-recognized that the miR-29 family exerts anti-fibrotic activity [36, 54–56]. The latest, exosome-mediated miR-29 metastasis attenuated muscle atrophy and renal fibrosis in a mouse model of unilateral ureteral obstruction [57]. Some scholars believe that fibrosis characterized by type-3 collagen hyperplasia hinders tendon repair or reversal of tendinopathy [58]. Recent work suggested the potential beneficial effect of miR-29a on tendinopathy. In a randomized, double-blind trial using the horse tendon model, Watts et al. improved early tendon injury with miR-29a local treatment [59]. Millar et al. reported that miR-29a regulated tissue remodeling in tendinopathy by targeting type-3 collagen [60]. After observing that HUMSC-Exos improved tendon healing, we further analyzed the underlying mechanism of the action using miRNA-seq technology. The data indicated that HUMSC-Exos had differentially higher expression of miR-29a-3p than their donors, whereas rat tendons treated with HUMSC-Exos showed elevated expression of miR-29a-3p. This suggested that the role of HUMSC-Exos in improving tendon healing was closely related to miR-29a-3p expression. To validate this hypothesis, we subsequently treated HUMSCs with specific agonist targeting miR-29a-3p to obtain engineered exosomes that specifically overexpressed miR-29a-3p, for the subsequent in vivo and in vitro assays. The results showed that HUMSC-Exos overexpressing miR-29a-3p further amplified the gain effects on tendon healing. Notably, in previous studies, topical miR-29a treatment alone reduced type-3 collagen level without altering the expression of type-1 collagen [59, 60]. However, our data showed that HUMSC-Exos overexpressing miR-29a-3p not only decreased type-3 collagen expression, but also increased the content of tendon markers such as COL1A1, SCXA, and TNMD. One possible explanation might be that exosomes carried a collection of many miRNAs, of which miR-29a-3p is not the only one beneficial for tendon repair in potentially favorable miRNAs. This provides supporting evidence for the application of engineered exosomes to tendon healing.

In view of the results of the KEGG analysis, mTOR signaling was one of the most significant pathways in the differentially treated tendon tissues, which allowed us to focus on the mTOR pathway in this investigation. We employed WB to verify tendon tissues with or without HUMSC-Exos intervention and found that the p-mTOR level was significantly elevated in HUMSC-Exo-treated tendons. This suggested that HUMSC-Exos might improve tendon healing by activating the mTOR pathway. It is well known that the mTOR signaling regulates protein synthesis, metabolism, and cell growth [61]. Liu et al. reported that in the rat rotator cuff injury model, the AKT/mTOR signaling pathway was downregulated after crosscutting the tendon, which resulted in reduced protein synthesis, and ultimately, muscle atrophy [46]. Cong et al. found that the inhibition of the mTOR signaling pathway significantly reduced the production of type-1 collagen and impaired the ability of MSCs to differentiate into tendons. In addition, they observed tendon defects and type-1 collagen reduction in tendon-specific mTOR-deficient mice. These results indicated that the AKT-mTOR axis might be a key mediator of tendon differentiation [45]. We found in vitro that HUMSC-Exos overexpressing miR-29a-3p significantly increased the expression of tendon markers in TDCSs, meanwhile, the level of p-mTOR also increased significantly. Notably, rapamycin, a kind of mTOR pathway inhibitor, inhibited the above performances significantly. These results suggested that HUMSC-Exos might improve tendon healing by activating the mTOR pathway. This was consistent with the results of previous research, where the long-term use of the mTOR inhibitor rapamycin delayed the aging process of the mouse tendon ECM [62]. Further, we identified PTEN as the direct target of miR-29a through the TargetScan prediction and dual luciferase assay and demonstrated the downregulation of PTEN expression in HUMSC-Exos treated TDSCs, which was reversed by the miR-29a antagonist. Given the evidences that PTEN is one of the critical negative regulators of mTOR signaling, the promoting effects of HUMSC-Exos on mTOR signaling could thus be explained. Accordingly, the phosphorylation of AKT was significantly elevated after HUMSC-Exos treatment and act as the upstream inducing event for mTOR phosphorylation, whereas miR-29a antagonist produced the opposite results. In line with these results, PTEN/AKT signaling was also reported to be closely related to the TDSCs senescence and tendon atrophy [63], while decreasing PTEN/AKT signaling yielded benefits for inhibiting TDSCs adipogenesis and tendon fatty infiltration [64]. Thus, our results in this study collectively proposed that HUMSC-Exos might enhanced the tenogenic potential of TDSCs through modulating PTEN/AKT/mTOR pathway by transferring miR-29a.

Substantial evidences show that TGF-β1 is one of the important downstream effectors of the mTOR signaling pathway, which is also a master regulator of tissue remodeling [45, 65, 66]. In this study, we found that HUMSC-Exos significantly elevated the TGF-β1 production in TDSCs, and HUMSC-Exos overexpressing miR-29a-3p further enhanced this effect in vitro. Further experiments showed that the TGF-β1 inhibitor (SB-431542) inhibited HUMSC-Exo-mediated changes in DCN, SCXA, and collagen expression levels significantly. Notably, rapamycin also inhibited the change in p-mTOR expression mediated by HUMSC-Exos. These results suggested that the PTEN/mTOR/TGF-β1 signaling cascade might be a key signal in HUMSC-Exos mediated delivery of miR-29a-3p for promoting tendon healing. Our findings above indicate that HUMSC-Exos and engineered HUMSC-Exos will have great potential in tendon defect regeneration in preclinical and clinical application.

## Conclusions

In summary, our findings confirmed that HUMSC-Exos promoted tendon healing. MiR-29a-3p might be a key miRNA for HUMSC-Exos to exert beneficial effects on tendon healing. Finally, the PTEN/mTOR/TGF-β1 signaling cascade might serve as a key mediator of HUMSC-Exos to improve tendon healing by delivering miR-29a-3p. The findings provide a theoretical basis for the application of stem cell-derived engineered exosomes in tendon injury regeneration.

## Data Availability

All data generated or analyzed during this study are included in this article.

## Abbreviations

HUMSC: Human umbilical cord mesenchymal stem cell
HUMSC-Exos: Human umbilical cord mesenchymal stem cell-derived exosomes
TDSC: Tendon-derived stem cell
TGF-β1: Transforming growth factor-β1
ECM: Extracellular matrix
MiRNA/miR: MicroRNA
SCXA: Scleraxis
MKX: Mohawk
DCN: Decorin
TNMD: Tenomodulin
UTS: Ultimate tensile strength
NTA: Nanoparticle tracking analysis
PTEN: Phosphatase and tensin homolog
